# Supplementary material for: The association between competitor level and the physical preparation practices of amateur boxers
Source: PLoS One. 2021 Sep 27;16(9):e0257907. doi: 10.1371/journal.pone.0257907 (PMC8475992; doi:10.1371/journal.pone.0257907)
Supplement: S1 Table — Survey questions. (DOCX) [file pone.0257907.s001.docx]

|  | Question | Question format |
| --- | --- | --- |
| **Boxer background and experience** | | |
| Q1 | Age? | Drop-down |
| Q2 | Sex? | Drop-down |
| Q3 | Boxing Club? | Short answer text |
| Q4 | Please select the category of boxer 9skill level) that applies to you? | Multiple choice |
| Q5 | Competition weight (kg)? | Short answer text |
| Q6 | How many years have you trained in boxing/ | Multiple choice |
| Q7 | Highest level you have competed at? | Multiple choice |
| **Access to performance staff** | | |
| Q1 | Do you have access to a strength & conditioning coach or sport scientist? | Multiple choice |
| **Physical Training** | | |
| Q1 | In relation to your training activities, select all that applies in a typical week. | Tick box grid with ‘other’ option |
| Q2 | Considering the schedule, please give an estimate of weekly training hours. | Drop-down |
| Q3 | In relation to your training activities, select all that applies in the week leading up to a bout. | Tick box grid |
| Q4 | Considering the schedule, please give an estimate of weekly training hours. | Drop-down |
| Q5 | Are your boxing sessions adapted for each individual boxer, or are they typically group themed? | Multiple choice |
| Q6 | Boxers perceptions of specific fitness adaptations to circuit training. | Checkboxes |
| Q7 | Please rank the following physical qualities (Strength, speed, power, aerobic fitness, anaerobic fitness) in order of importance to you as a boxer. | Multiple choice grid |
| **Monitoring** | | |
| Q1 | Do you use any of the scientific methods below to measure intensity of single sessions? | Checkboxes with ‘other’ option |
| **Testing** | | |
| Q1 | Do you perceive regular fitness testing to be beneficial to boxing performance? | Multiple choice |
| Q2 | Do you regularly use any of the scientific tests or protocols below to assess your fitness and/or the effectiveness of training? | Checkboxes with ‘other’ and ‘no’ options |
| **Perceptions of scientific support and potential barriers** | | |
| Q1 | Using the scale below, how much do you feel sport science and strength and conditioning support can improve your physical preparation? | Likert scale |
| Q2 | Please select the potential barriers to you as a boxer, in implementing a more evidence-based approach to your physical preparation? | Checkboxes with ‘other’ and ‘none’ options |

**S1 Table: Physical Preparation Practices of Amateur Boxers Survey**

Survey questions

Q = Question number.
