# Supplementary material for: The association between competitor level and the physical preparation practices of amateur boxers
Source: PLoS One. 2021 Sep 27;16(9):e0257907. doi: 10.1371/journal.pone.0257907 (PMC8475992; doi:10.1371/journal.pone.0257907)
Supplement: Attachment — Submitted filename: Reply letter to reviewer comments.docx [file pone.0257907.s003.docx]

Manuscript PONE-D-21-10978 R2

**Reply letter to comments on the manuscript**

Dear Emiliano Ce,

Thank you to you and your editorial team for the opportunity to submit a revised version of the manuscript. We are delighted that we have adequately responded to all reviewer comments. We would like to send our appreciation once again to the two reviewers for their time and expertise. In relation to the single suggestion provided by reviewer 2 that required minor revision, this has been addressed below.

Once again, thank you.

Reviewer 2

1. Since the sample is composed by either males and females and the effort perceptions or structure of intensity of the training could be different in the two sexes, did you tried or would you try to adjust the analysis by sex to highlight if the sex variable is significant in your study? i strongly suggest to perform this analysis. on the contrary, I suggest to add this point in the limitation section and consider sex differences in further studies.

**Authors reply – The authors thank reviewer 2 for this suggestion. We had initially considered analysing the differences in practices by sex; however, due to the small sample of female participants, this analysis was not possible. The authors definitely agree that this is an interesting area and one worth investigating, particularly as female boxing continues to grow in popularity. The authors have now included this as a limitation, with a recommendation for further research to aim for a more proportionate sample size of males and females, in order to perform this analysis. Please see below.**

**“Future research may wish to aim for a more proportionate sample of males and females, in order to assess potential differences in the physical preparation practices between sexes.”**
